# The Neurobiology of Dual Diagnosis: Investigating the Shared Pathophysiology of Alcohol Use Disorder and Major Depressive Disorder

**DOI:** 10.1192/j.eurpsy.2026.10455

**Published:** 2026-07-31

**Authors:** A. H. I. Abu Shehab, Ș. L. Burlea, I. Chiscop, A. A. Ș. Baltă, R. G. C. Cobzaru, A. Bulgaru Iliescu, D. C. Voinescu, A. Ciubară

**Affiliations:** 1 Psychiatry, “Elisabeta Doamna” Psychiatry Hospital, Galați, Galati; 2 Psychiatry, “Grigore T. Popa” University of Medicine and Pharmacy (UMF), Iași, Iasi; 3 Oral & Maxillofacial Surgery, “Sf. Apostol Andrei” Emergency Clinical County Hospital, Galați; Faculty of Medicine & Pharmacy, “Dunărea de Jos” University of Galați; 4 Medical/Clinical Department & Doctoral School of Biomedical Sciences, Faculty of Medicine and Pharmacy, “Dunărea de Jos” University of Galați, Galati; 5 Microbiology, “Grigore T. Popa” University of Medicine and Pharmacy (UMF), Iași; 6 Morphofunctional Sciences, County Emergency Clinical Hospital “St. Spiridon” Iași; “Grigore T. Popa” University of Medicine and Pharmacy (UMF), Iași, Iasi; 7 Clinical Medical/Rheumatology Compartment, “Sf. Apostol Andrei” Emergency Clinical County Hospital, Galați; Faculty of Medicine & Pharmacy, “Dunărea de Jos” University of Galați; 8 Psychiatry, Faculty of Medicine and Pharmacy, “Dunărea de Jos” University of Galați; “Elisabeta Doamna” Psychiatry Hospital, Galați, Galati, Romania

## Abstract

**Introduction:**

The co-occurrence of Alcohol Use Disorder and Major Depressive Disorder represents a significant clinical challenge, with lifetime comorbidity rates of 20.5%. This dual diagnosis is associated with increased severity, poorer treatment outcomes, and higher suicide risk compared to either disorder alone.

**Objectives:**

To synthesize current research on the shared neurobiological mechanisms underlying comorbidity and identify common pathophysiological pathways that contribute to dual diagnosis vulnerability.

**Methods:**

Comprehensive literature review of peer-reviewed research examining neurobiological mechanisms in AUD-MDD comorbidity, including neurotransmitter systems, stress response pathways, neuroimmune processes, and genetic factors. Sources included PubMed, PMC, and specialized psychiatric databases from 2010-2025.

**Results:**

Evidence supports a shared vulnerability model involving multiple interconnected systems: (1) Dysregulation of dopaminergic reward pathways contributes to both alcohol reinforcement and anhedonia; (2) Serotonergic dysfunction underlies mood disturbances in both conditions; (3) GABAergic system alterations mediate anxiety and withdrawal symptoms; (4) HPA axis dysfunction shows biphasic patterns with initial hyperresponsiveness followed by hyporesponsiveness; (5) Neuroimmune activation through alcohol-induced gut permeability triggers neuroinflammation; (6) Genetic studies identify shared loci on chromosome 1 predisposing to both disorders; (7) Epigenetic modifications mediate gene-environment interactions.

**Conclusions:**

The comorbidity of Alcohol Use Disorder and Major Depressive Disorder reflects shared neurobiological vulnerability rather than simple causal relationships. Common pathophysiological mechanisms create interconnected cycles of dysfunction across neurotransmitter, stress, and immune systems. These findings support integrated treatment approaches targeting shared pathways and highlight the need for personalized interventions based on neurobiological profiles.

**Disclosure of Interest:**

None Declared

